# Caplacizumab: frequent local skin reactions

**DOI:** 10.1007/s00277-020-04260-7

**Published:** 2020-09-30

**Authors:** Jessica Kaufeld, Paul T. Brinkkoetter, Adrian Schreiber, Wolfram J. Jabs, Markus Bieringer, Heike Bruck, Jan Menne, Linus A. Völker

**Affiliations:** 1 Department of Nephrology and Hypertension, Medical School Hannover, Hannover, Germany; 2 Department II of Internal Medicine and Center for Molecular Medicine Cologne (CMMC), Faculty of Medicine and University Hospital Cologne, University of Cologne, Kerpener Str. 62, D-50937 Cologne, Germany; 3 Cologne Cluster of Excellence on Cellular Stress Responses in Ageing-Associated Diseases (CECAD), Cologne, Germany; 4 Charité - Universitätsmedizin Berlin, Department of Nephrology and Intensive Care Medicine, Berlin, Germany, and Experimental and Clinical Research Center, Charité, Max Delbrück Center for Molecular Medicine in the Helmholtz Association, Berlin, Germany; 5 Department of Nephrology, Vivantes Klinikum im Friedrichshain, Berlin, Germany; 6 Department of Cardiology and Nephrology, Helios Klinikum Berlin-Buch, Berlin, Germany; 7 Department of Nephrology, Helios Klinikum Krefeld, Krefeld, Germany

**Fig. 1:**
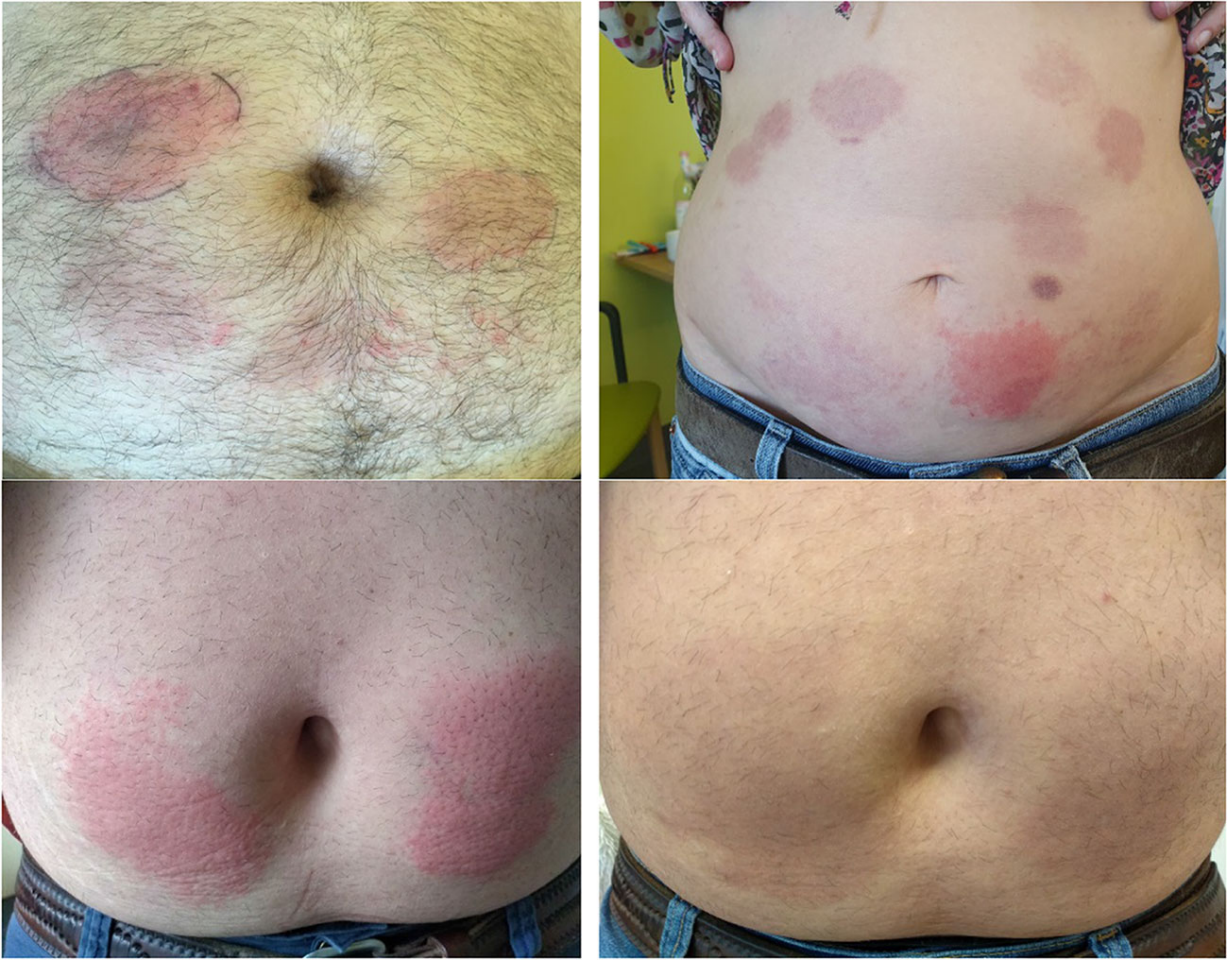
Local skin reaction with different time points after injection (upper panel). It takes about 7–14 days before lesions have disappeared (lower panel)

Dear Editor,

Caplacizumab (Cablivi®) is a novel anti-von Willebrand factor (vWF) nanobody approved for the treatment of acquired thrombotic thrombocytopenic purpura (aTTP) [1]. A single-dose (10 mg) caplacizumab is given intravenously before starting plasma exchange (PEX), followed by daily (10 mg) SC injections for up to 30 days after last PEX. We collected clinical data of 60 patients between 2018 and 2019 [2, 3]. In 7 cases (> 10%), we observed local injection site reactions appearing 4 days to 4 weeks after the start of the caplacizumab treatment. These skin reactions caused great uncertainty leading to an altered, off-label management in 5 cases and might interfere with the patients’ treatment outcome. The skin reactions presented as erythema with hyperreactivity around the injection site with hyperemia and itching (Fig. 1). The findings developed sooner and more pronounced with subsequent injections even at different sites. There were no significant changes in the eosinophilia count, IgE levels, or C-reactive protein. Importantly, the reactions had no measurable impact on drug efficacy as vWF activity remained suppressed. The local reactions did not respond to anti-histaminic or high-dose glucocorticoid treatment. All patients were still on a steroid-tapering regime with dosages ranging from 12.5 to 80 mg/day prednisolone. In one patient, the caplacizumab dosing was not altered but prednisolone increased to 30 mg/day. In two other patients, caplacizumab was first continued and later extended to a q2 dosing regimen with daily prednisolone (30 mg). In another patient, caplacizumab was briefly interrupted before being continued in an unchanged alternate dosing regimen and 50 mg of prednisolone in addition to each injection. Caplacizumab was discontinued in 3/6 patients. In one of these 3 patients, an aTTP relapse occurred after stopping the treatment, and caplacizumab was restarted on a q2 dosing regimen together with prednisolone (12.5 mg/day). After discontinuation of caplacizumab, the skin reactions slowly disappeared. In patients with ongoing treatment, the skin reactions peaked before slowly resolving over several weeks independent of any of the abovementioned measures. All patients had a full recovery from aTTP.

In the HERCULES trial, urticaria and local injection site pain were reported as an adverse event in 16.9% and 1.4% of patients in the caplacizumab arm and 6.8% and 5.5% in the placebo arm [1] potentially as a reaction to the fresh frozen plasma and the PEX. [4]. In our patients, the erythema always occurred several days/weeks after the cessation of PEX. Pre-existing allergies were not reported.

One excipient in the formulation of Cablivi is Polysorbate 80 besides sucrose, citric acid, and sodium citrate. Polysorbates are often added to stabilize new biotherapeutics such as new formulations of erythropoeitin or PCSK9i [5, 6]. Polysorbates have the potential to activate complement and may cause acute hypersensitivity reactions as well as anaphylactoid reactions and local skin reactions [7]. Further studies are required to delineate the role of Polysorbate 80 and a causal relationship for the observed symptoms.

We suggest monitoring patients closely for evolving local skin reactions and that caplacizumab should only be prematurely discontinued in cases with systemic side effects (hypotension, wheezing, generalized rash). Extending the dosing interval to every other day using vWF activity measurement to guide the treatment appears to be a safe and feasible therapeutic option in patients with a local skin reaction [3].
